# Intima Sarcoma of the Pulmonary Artery Mimicking Takayasu Disease

**DOI:** 10.1155/2011/510708

**Published:** 2011-08-23

**Authors:** C. Belge, I. Renckens, R. Van Puijenbroek, W. Wuyts, B. Meyns, M. Delcroix

**Affiliations:** ^1^Department of Respiratory Medicine, University Hospitals Leuven, 3000 Leuven, Belgium; ^2^Department of Anatomopathology, University Hospitals Leuven, 3000 Leuven, Belgium; ^3^Department of Respiratory Medicine, Sint-Franciskus Ziekenhuis Hospital-Heusden-Zolder, 3550 Heusden-Zolder, Belgium; ^4^Department of Cardiac Surgery, University Hospitals Leuven, 3000 Leuven, Belgium

## Abstract

Pulmonary artery intima sarcoma is an uncommon but fatal tumor, which often masquerades chronic thromboembolic pulmonary hypertension (CTEPH) and in the present case Takayasu arteritis. Pulmonary arterial pressure is mildly elevated in the presence of extensive proximal lesions. A parenchyma thin-walled cavitary lesion may be a sign of pulmonary extravasation of the tumor.

## 1. Case Report

A 31-year-old woman was referred to our hospital for persistent dyspnea on exertion (New York Heart Association, NYHA, class 2) and CTEPH. She had a history of hookworm infection, and 4 years ago she had an episode of acute chest pain and shortness of breath. A second episode occurred 3 months ago, being interpreted as acute pulmonary embolism precipitated by the combination of pill and smoking.

Clinical examination revealed clubbing, a prominent pulmonary component of the second heart sound and a left parasternal systolic ejection murmur. Peripheral arterial pulsations were normal, there was no carotid arteries murmur, and blood pressure was the same on both arms (99/60 mmHg). The remainder of the examination was unremarkable. 

**Figure 1:**
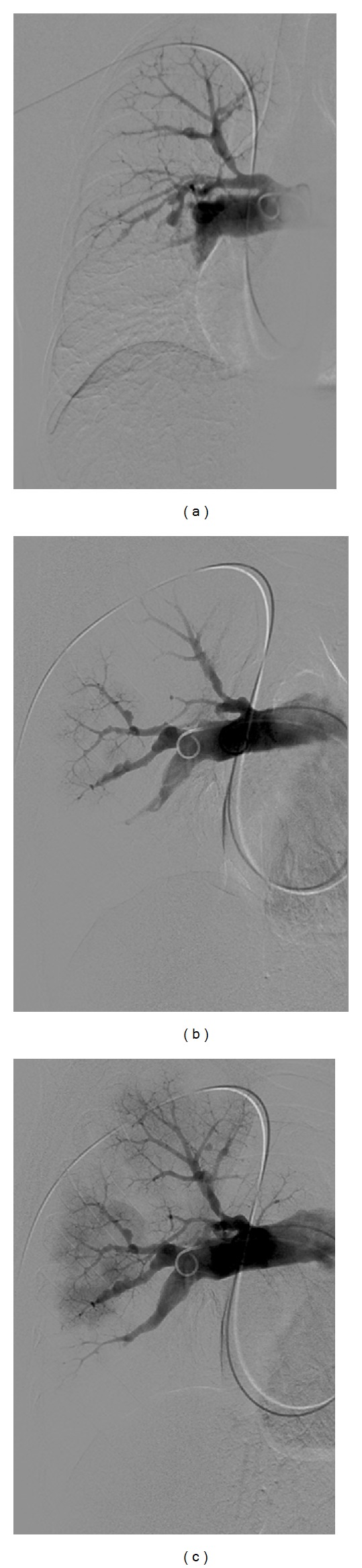
Pulmonary angiography suggestive for chronic thromboembolic pulmonary hypertension (CTEPH) with unexplained multiple dilatations of the branches of the right upper lobar artery.

**Figure 2:**
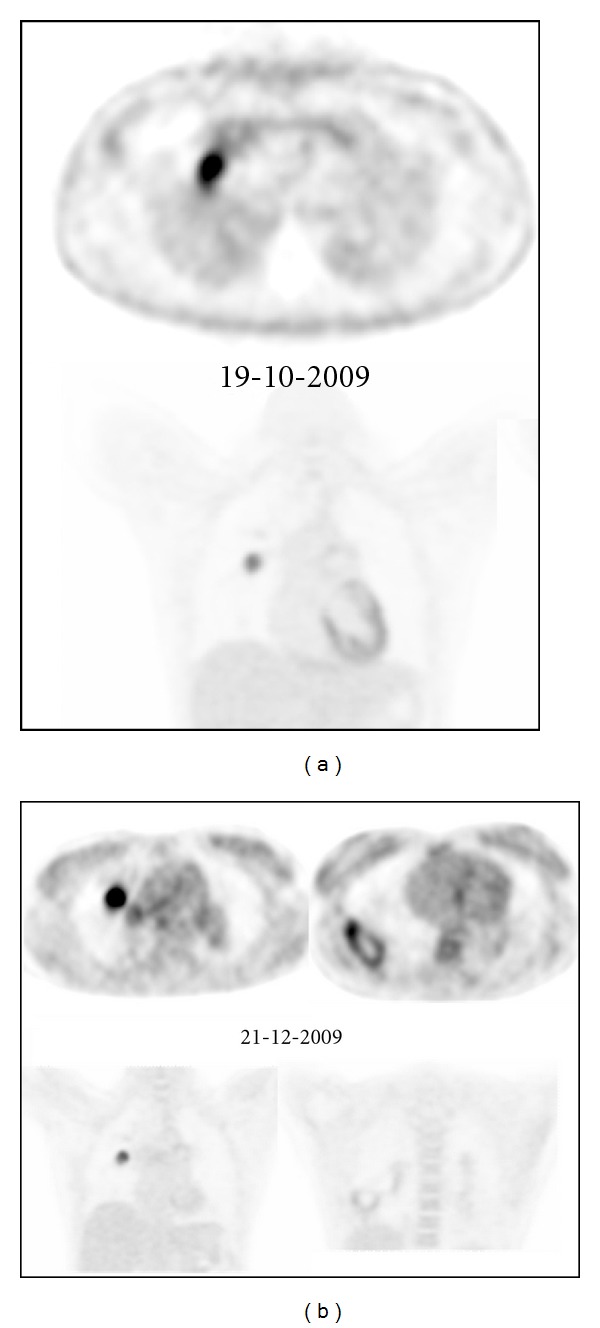
(a) Initial PET scan. Metabolic active lesions in the right upper lobe which diameter increased over time, with appearance of a metabolically active cavity in the right lower lobe. (b) Follow-up PET scan after 2 months of oral corticosteroids. The diameter of the metabolic active lesions in the right upper lobe increased over time, with appearance of a metabolically active cavity in the right lower lobe.

Routine laboratory examination did not show any signs of inflammation. Electrocardiogram was normal. Echocardiogram showed a thickened mitral valve, a tricuspid regurgitation of 3/4 with an estimated systolic pulmonary arterial pressure (PAP) of 60 mmHg, and a markedly dilated, mildly hypocontractile right ventricle. Right heart catherisation showed a mean PAP of 28 mmHg, a cardiac index of 2.91 L/min/m^2^, and a total pulmonary vascular resistance of 450 dyne.sec.cm^−5^. Exercise capacity was impaired as shown by a 6 min walking distance of 553 meters (69% of predicted) with desaturation from 96 to 90% and by an ergospirometry showing a peak oxygen consumption of 22 ml/min/kg with desaturation from 97 to 83%. Lung function was normal except for a low diffusion capacity of 46%. Chest X-ray showed enlarged pulmonary arteries with a right parahilar nodule, perfusion scan multiple lobar and segmental defects, and pulmonary angiography amputations of the right lower lobe and left upper lobe arteries, with multiple aneurysmal dilatations of the branches of the right upper lobe artery (Figure 1). CT of the chest confirmed the presence of a nodule in the right upper lobe, which was metabolically active on PET scan (Figure 2(a)).

**Figure 3:**
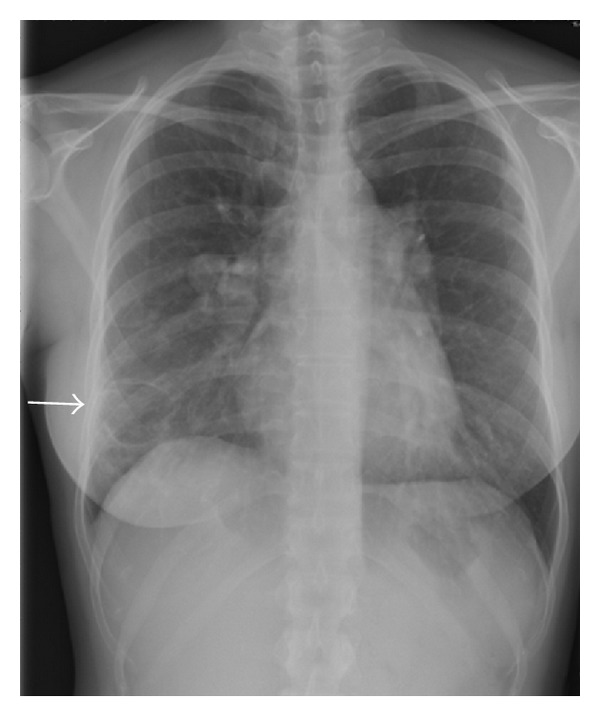
Follow-up chest X-ray shows a thin-walled cavitary lesion in the right lower lobe (white arrow).

**Figure 4:**
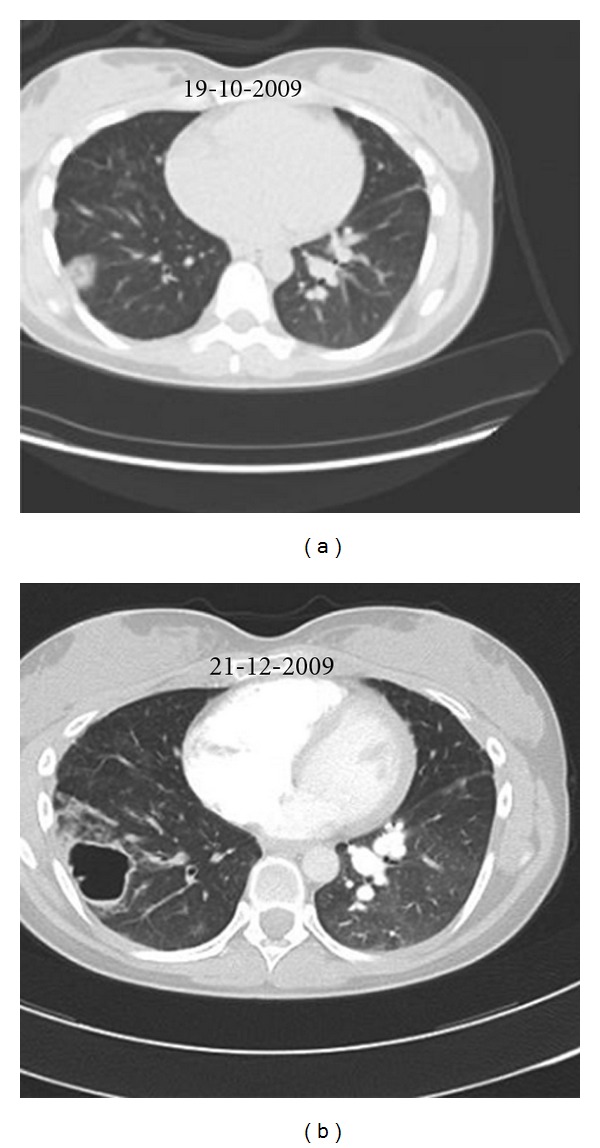
CT thorax shows an initial lung infiltrate (a) evolving into a large thin-walled cavitary lesion in the right lower lobe (b).

**Figure 5:**
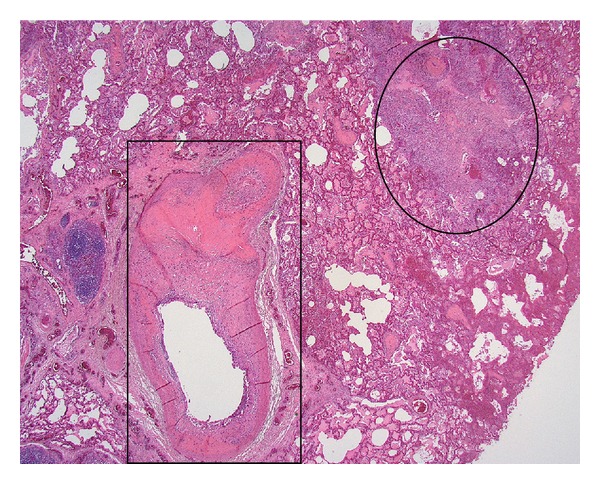
This section of the lung by autopsy showed a dilated vessel with tumor (intima sarcoma, rectangle) and extravascular extension of the tumor (circle). (Hematoxylin and eosin, ×12.5).

In our patient, a 4-item differential diagnosis was discussed: (1) CTEPH, but angiography was somewhat atypical and pulmonary hypertension was mild; (2) Takayasu arteritis [1], but without systemic inflammation or proximal systemic artery disease at CT, MRI, and PET scans; (3) mycotic pulmonary aneurysms [2], again without systemic inflammation or chronic infection; (4) pulmonary artery intima sarcoma, but without proliferative process in central pulmonary arteries. After local as well as international multidisciplinary consultations, we initially rejected the diagnosis of pulmonary intima sarcoma and tackled the diagnosis of Takayasu disease by treating the patient with high doses of oral corticosteroids combined with ongoing anticoagulation. After 2 months of therapy, evolution was characterized by clinical deterioration with appearance of cough, brown and sometimes bloody expectorations and intermittent fever, by a slightly increased CRP (19 mg/L, <5), and by progression of the lesions on Chest X-ray (Figure 3), CT (Figure 4), and PET (Figure 2(b)) scans. The right upper lobe nodular lesion was enlarged, while a thin-walled large cavity had replaced a previously known pleural based condensation in the right lower lobe. A bronchoalveolar lavage showed aspergillus fumigates colonization and voriconazole was added to the therapy while corticosteroids were downtitrated. Retrospectively, we found evidence for infiltrates followed by infarction/cavitation also in the upper segment of the left lower lobe. As intima sarcoma of the pulmonary artery with distal embolisation was the most probable diagnosis, it was decided to perform a pulmonary endarterectomy later completed by lung resection and/or chemotherapy with tyrosine kinase inhibitors according to cell type. While the macroscopic aspect of the surgical material was suggestive for CTEPH, histopathology confirmed the diagnosis of undifferentiated intima sarcoma. In our patient the disease progressed unfortunately very rapidly after surgery with ICU readmission on day 9 because of chest pain, severe progressive hypoxemia, and hemoptysis. As CT scan showed multiple infiltrates and recurrent obstruction of the right lower lobe artery it was decided to withhold therapy. Autopsy showed (i) residual intima sarcoma in the peripheral branches of both pulmonary arteries, with complete or partial vessel wall invasion and extravascular extension (Figure 5), (ii) a myxoid nodular mass, 3 cm in diameter, in the right middle lobe, (iii) a large “cavity” with myxoid center, more specifically formed by oedematous tumoral tissue forming a lattice-like network responsible for radiolucency, in the right lower lobe. There were no signs of infection or aspergillar invasion.

## 2. Discussion

Pulmonary artery intima sarcoma, first described by Mandelstamm in 1923 [3], is a rare but potentially lethal tumour. Fewer than 250 cases are described in the literature. The prognosis of patients with primary sarcoma of the pulmonary artery is very poor. The median survival time is 1.5 months and can be prolonged to 10 months by surgical tumor excision [4]. Extensive tumor resection with wide security margins and pulmonary artery reconstruction has been proposed but pulmonary endarterectomy [5] has also been performed because the tumor is often limited to the intima. There appears to be no consensus regarding the benefit of adjuvant chemotherapy [6].

This case illustrates that intima sarcoma is often initially misdiagnosed, usually because it mimics CTEPH, but in this case because it mimics large-vessel arteritis. It draws attention to the need to raise differential diagnosis in patients with atypical features of CTEPH such as clubbing [5], unilateral distribution of a massive perfusion defect [7], or aneurysmal dilatations of the pulmonary arterial branches and lung cavities as in the present case. If pulmonary artery sarcoma presents in the form of an acquired pulmonary artery aneurysm, it has to be differentiated from Behçet's disease, primary lung tumors, metastatic disease, and mycotic aneurysms [2]. To the best of our knowledge this is the first report of a thin-walled cavity, while few cases of thick-walled cavities have already been reported in patients with pulmonary artery sarcoma [8, 9].

In conclusion, pulmonary artery sarcoma is an uncommon but fatal tumor which often masquerades CTEPH. In the present case it presents more like arteritis.

Extensive tumor resection with wide security margins and pulmonary artery reconstruction is the treatment of choice when the patient is scheduled for elective surgery but pulmonary endarterectomy can also be performed. The sequence infiltration-(infarction)-cavitation may be a sign of extravascular extension into the lung parenchyma.
